# Gene expression profiling reveals activation of the FA/BRCA pathway in advanced squamous cervical cancer with intrinsic resistance and therapy failure

**DOI:** 10.1186/1471-2407-14-246

**Published:** 2014-04-08

**Authors:** Ovidiu Balacescu, Loredana Balacescu, Oana Tudoran, Nicolae Todor, Meda Rus, Rares Buiga, Sergiu Susman, Bogdan Fetica, Laura Pop, Laura Maja, Simona Visan, Claudia Ordeanu, Ioana Berindan-Neagoe, Viorica Nagy

**Affiliations:** 1 The Oncology Institute "Prof Dr. Ion Chiricuta", 34-36 Republicii street, 400015 Cluj-Napoca, Romania; 2 Iuliu Hatieganu, University of Medicine and Pharmacy, 8 Babes street, 400012 Cluj-Napoca, Romania; 3 Faculty of Veterinary Medicine, University of Agricultural Sciences and Veterinary Medicine, 3-5 Calea Manastur street, 400372 Cluj-Napoca, Romania

**Keywords:** FANCD2, RAD51, BRCA1, BRIP1, Cervical cancer, Microarray, Treatment response

## Abstract

**Background:**

Advanced squamous cervical cancer, one of the most commonly diagnosed cancers in women, still remains a major problem in oncology due to treatment failure and distant metastasis. Antitumor therapy failure is due to both intrinsic and acquired resistance; intrinsic resistance is often decisive for treatment response. In this study, we investigated the specific pathways and molecules responsible for baseline therapy failure in locally advanced squamous cervical cancer.

**Methods:**

Twenty-one patients with locally advanced squamous cell carcinoma were enrolled in this study. Primary biopsies harvested prior to therapy were analyzed for whole human gene expression (Agilent) based on the patient’s 6 months clinical response. Ingenuity Pathway Analysis was used to investigate the altered molecular function and canonical pathways between the responding and non-responding patients. The microarray results were validated by qRT-PCR and immunohistochemistry. An additional set of 24 formalin-fixed paraffin-embedded cervical cancer samples was used for independent validation of the proteins of interest.

**Results:**

A 2859-gene signature was identified to distinguish between responder and non-responder patients. ‘DNA Replication, Recombination and Repair’ represented one of the most important mechanisms activated in non-responsive cervical tumors, and the ‘Role of BRCA1 in DNA Damage Response’ was predicted to be the most significantly altered canonical pathway involved in intrinsic resistance (p = 1.86E-04, ratio = 0.262). Immunohistological staining confirmed increased expression of BRCA1, BRIP1, FANCD2 and RAD51 in non-responsive compared with responsive advanced squamous cervical cancer, both in the initial set of 21 cervical cancer samples and the second set of 24 samples.

**Conclusions:**

Our findings suggest that FA/BRCA pathway plays an important role in treatment failure in advanced cervical cancer. The assessment of FANCD2, RAD51, BRCA1 and BRIP1 nuclear proteins could provide important information about the patients at risk for treatment failure.

## Background

Cervical cancer, the third most commonly diagnosed cancer in women, with 529,800 cases in 2010 [1], represents a major problem in oncology due to treatment failure and distant metastasis. More than 85% of cervical cancers are diagnosed every year in developing countries, and approximately 90% of overall deaths occur in these countries. If detected at an early stage, cervical cancer represents one of the most successfully treated cancers. Unfortunately, because of the lack of screening programs in developing countries, cervical cancer is predominantly detected in advanced stages (IIB-IIIB). About half of the patients with advanced cervical cancer will develop recurrence or metastasis in the first 2 years after completion of therapy.

Although new anticancer drugs are constantly being developed, overcoming drug resistance is still a challenge. Therefore, there is an urgent need to identify new prognostic factors that could distinguish between patients with unfavorable prognoses from others with better prognoses.

Almost half of patients present baseline resistance (intrinsic resistance), and a large proportion of the remaining half will develop resistance during treatment (acquired resistance) [2]. Intrinsic resistance is often complex and occurs through several mechanisms, depending on the therapy regimen. The treatment for pre-invasive lesions is generally based on surgery; for invasive cervical cancers, the treatment is based on surgery and/or radiation and cisplatin-based chemotherapy [3]. The chemoradiotherapy treatment produces DNA double-strand breaks (DSBs), which is considered to be the most lethal form of DNA damage. DSBs are caused by radiation and platinum compounds based chemotherapy but also could be produced by endogenous damage, such as that caused by reactive oxygen species and collapsed replication forks. DNA damage induces a series of molecular responses that are responsible for the maintenance of genome integrity [4]. Deficiencies in DSB response and repair could represent important events for intrinsic resistance.

The diagnosis of baseline resistance in individual patients could improve the cancer treatment by the avoidance of inefficient therapy. Gene expression studies have been conducted across many tumor types to investigate the patterns of genes involved in intrinsic resistance. In cervical cancer, relatively few studies have been focused on identifying baseline resistance to chemoradiotherapy [5-7]. Therefore, the aim of our study was to investigate the specific pathways and molecules responsible for baseline therapy failure in locally advanced squamous cervical cancer.

## Methods

### Sample collection

Patient samples and clinical data with end points were obtained from the Departments of Radiotherapy and Pathology of The Oncology Institute ‘Prof. Dr. I. Chiricuta’, Cluj-Napoca, Romania. This study was approved by the ethics committee of The Oncology Institute ‘Prof. Dr. Ion Chiricuta’. All patients gave informed consent in accordance with the Declaration of Helsinki.

Twenty-one patients with locally advanced squamous cell carcinoma (FIGO stage IIB-IIIB) were enrolled in the genomics study. A tissue fragment from a primary biopsy and a cervical lavage specimen were harvested from each patient prior to initiation of the therapy. Tissue samples were stored in liquid nitrogen until use for RNA extraction.

Corresponding formalin-fixed paraffin-embedded (FFPE) tissue samples were used for protein validation. Moreover, an additional set of 24 FFPE samples was used for independent immunohistochemistry validation of the data. All patients in the validation and study groups had the same including criteria. The clinical and histopathological characteristics of the patients included in this study are presented in Table 1.

**Table 1:** Baseline characteristics of the patients in the genomics study and IHC validation group

**Characteristics**	**Genomics study group (n = 21)**	**IHC validation group (n = 24)**
**Median age (range), years**	46 (27–73)	52 (28–62)
**Median tumor size (range), cm**	5 (2–8)	4 (2–7)
**Median hemoglobin (range), g/dl**	12.7 (7.9–14.4)	13.3 (10.2–14.9)
**FIGO stage**		
II B	10	8
III A	5	11
III B	6	5
**HPV subtype**		
HPV 16	16	19
Other high-risk*	3	3
Negative	2	2
**Treatment response**		
CR	12	15
NCR	9	9

*other high-risk in study group: 33,58,73.

other high-risk in validation group: 31,45,58.

### The therapy schedule

The patients were treated with concomitant chemoradiotherapy (CRT) associated or not with surgery. The radiotherapy protocol includes external beam radiotherapy (EBRT) to the pelvis delivered by a linear accelerator at 15MV for a dose of 46 Gy/23 fractions and a cervical boost given by intracavitary high-dose-rate (HDR) brachytherapy (BT) in a dose of 10 Gy/2 fractions. Cisplatin was administered concomitant with the radiotherapy as a radiosensitizer. At this dose, patients were evaluated and, according to tumor response, further of CRT (EBRT until 60 Gy concomitant with cisplatin and HDR BT until 20 Gy) or surgery (radical abdominal hysterectomy with pelvic lymphadenectomy) was decided. In our internal protocol, surgery was recommended, but not mandatory, being a patient’s option. The tumor response was clinically evaluated at 6 months after the end of the CRT treatment and was defined as complete response (CR) or non-complete response (NCR) (partial response and stable disease). For the patients that underwent surgery, the histopathological evaluation confirmed the clinical response.

### RNA extraction and purification

Tumor sections with a minimum of 70% tumor cells were harvested by macrodissection from primary biopsies of cervical cancers. Total RNA was extracted with TriReagent (Sigma-Aldrich) and purified using an RNeasy Mini kit (Qiagen) according to the manufacturer’s protocols. Extracted RNA was assessed for quality with a Lab-on-a-chip Bioanalyzer 2100 (Agilent Technologies). The RNA Integrity Number (RIN) and rRNA 28S/18S ratio were used to define the quality of the total RNA. The RNAs with RINs >7.5 and rRNA 28S/18S ratios >1.8 were used for further analysis. RNA concentrations were adjusted using a NanoDrop ND-1000 spectrophotometer (NanoDrop Technologies).

### HPV genotyping

Genomic DNA was extracted from 1 ml of cervical lavage using a High Pure DNA extraction kit (Roche). HPV genotypes, including 37 high- and low-risk genotypes, were identified with the Linear Array HPV Genotyping Test (Roche) according to the manufacturer’s protocol.

### Oligonucleotide microarray technology

Agilent oligonucleotide technology was used to measure gene expression changes in the samples of interest. Microarray probes (cRNA-Cy3) were synthesized from 200 ng of total RNA in two reaction steps using a one-color Agilent Low Input Quick Amp Labeling Kit according to the manufacturer’s instructions. All labeled cRNAs (Cy3) were purified using an RNeasy Mini kit (Qiagen) and were evaluated for quality control using a Nanodrop ND-1000 spectrophotometer. cRNAs with minimum yields of 1.65 μg and specific activities of 6 pmol/μl Cy3 per μg cRNA were selected for further analysis. After fragmentation to an average size of 60 – 100 nucleotides, each cRNA was hybridized for 17 hours at 65°C to whole-human-genome 4×44K microarray slides (product G4112F; Agilent) following the manufacturer’s protocol (Agilent Technologies). The slides were scanned with an Agilent G2505B US45102867 microarray scanner, and gridding was performed with Feature Extraction Software v.10.5.1.1.

The microarray data have been deposited in the NCBI Gene Expression Omnibus (GEO) repository under accession number GSE56363.

### Microarray data analysis

The microarray data, including median foreground and background intensities, flags and feature annotations, were imported into R/Bioconductor. The association between log2 values of background and foreground intensities across each array was estimated by computing Pearson correlation coefficients. Suitable R packages (arrayQualityMetrics, limma, marray) were used for quality control, normalization, filtering and data summarization. Between-array normalization was performed using the quantile normalization method. The median normalized signals were used for further data analysis. To reduce the number of non-informative features, the probes with saturated and non-uniform signals present in more than 15% of the samples were removed. Differentially expressed genes/sequences between non-responder and responder samples were selected using the moderated t-statistic. This method is an improvement over the standard t-statistic, as it allows elimination of the influence of random small within-group variance by sharing information across genes. The Benjamini and Hochberg method was used to adjust the p-values for multiple testing (adjusted p-value < 0.05). Only genes/sequences with at least a 1.5-fold change in expression between the studied groups were considered differentially expressed. The hierarchical clustering using Euclidean distances and Ward method was further performed to cluster the similarities in expression between genes/samples.

### Functional analysis

The dataset containing differentially expressed genes was uploaded into the Ingenuity Pathway Analysis (IPA) software (Ingenuity® Systems, http://www.ingenuity.com) and was associated with the biological functions and canonical pathways in the Ingenuity Knowledge Base. Fisher’s exact test (p < 0.05) was used to assess the significance of the associations between genes in the dataset and biological functions or canonical pathways. In addition, for canonical pathways, a ratio was computed between the number of molecules from the dataset and the total number of molecules in that pathway.

### Quantitative real-time PCR (qRT-PCR)

The First Strand cDNA Synthesis Kit (Roche) was used to reverse transcribe 200 ng of total RNA. Five microliters of 1:10 (v/v)-diluted cDNA was amplified in a final volume of 20 μl using a LightCycler 480 (Roche). The amplification was performed with 1 μM specific primers (Tib Molbiol) and a 0.2 μM specific hydrolysis probe from the Universal Probe Library (UPL). The primers and UPL probes were designed with Roche Applied Science software as follows: BRCA1 (NM_007294.3): F-ttgttgatgtggaggagcaa, R-ttgttgatgtggaggagcaa (UPL#11); BRCA2 (NM_000059.3): F-agcttactccggccaaaaa, R-ttcctccaatgcttggtaaataa (UPL#50); RAD51 (NM_001164269.1): F-tgagggtacctttaggccaga, R-cactgccagagagaccatacc (UPL#66); FANCD2 (NM_033084.3): F-cgacttgacccaaacttcct, R-tcctccaatctaatagacgacaact (UPL#9); BRIP1 (NM_032043.1): F-aatggcacttcatcaacttgtc, R-tggatgcctgtttcttagca (UPL#71); BLM (NM_000057.2): F-gatcagaaagcaccacccata, R-tcagccatggtgtcacattc (UPL#34); and 18S rRNA (NR_003286.2): F-gcaattattccccatgaacg, R- gggacttaatcaacgcacgc (UPL#48). Thermal cycling conditions were set as follows: activation at 95°C for 10 minutes; followed by 40 cycles of amplification, including denaturation at 95°C for 15 seconds, annealing at 55°C for 20 seconds and extension at 72°C for 1 second; followed by a cooling step at 40°C for 30 seconds. The relative expression levels of target genes (NCR vs. CR) were calculated using the ΔΔCt method [8] after normalizing to 18S housekeeping gene.

### Immunohistochemistry (IHC)

Immunohistochemistry was performed on FFPE 4-μm thick tissue sections, using a standard protocol. Following deparaffinization and rehydration of the tissue sections, antigen retrieval was performed for 20 minutes in 0.01 M citrate buffer (pH 6.0) using the boiling process (pressure cooker). Endogenous peroxidase was blocked with H_2_O_2_ (3%). Blocking of the nonspecific reactions was performed using the Novocastra Protein block™ solution. The sections were incubated 30 minutes with primary antibodies at room temperature in a humid chamber. The immunohistochemical staining was performed using the following dilutions for the primary monoclonal antibodies: 1:400 for BRCA1 (BioVision Inc., OH, USA, clone#3364-100), 1:200 for BRCA2 (Covalab, Cambridge, UK, clone pab0457-0), 1:200 for FANCD2 (Thermo Pierce Biotechnology Inc., IL, USA, clone PA1-16548), 1:20 for Rad51 (Thermo Pierce Biotechnology Inc., IL, USA, clone MA5-14416) and 1:300 for BPRIP1 (Abcam, Cambridge, UK, product number ab151509). Sections were sensitized using Post Primary Block™, and then incubated with NovoLink™ polymer containing the secondary antibody. The peroxidase reaction was developed using diamino-benzidine tetrachloride (DAB) as chromogen. Sections were counterstained with hematoxylin.

The IHC staining was automatically assessed using the ImmunoRatio free web-based application [9]. The application is conceived for automated image analysis of immunohistochemical nuclear staining like estrogen receptor (ER), progesterone receptor (PR), or Ki-67. Briefly, for every case 3 different representative images of immunostained sections were taken using a CX41 Olympus microscope coupled with a high resolution video camera AV5100M (MegaVideo IP camera, Arecont Vision). The application performs the segmentation of brown (DAB-colored), and hematoxylin-stained nuclei, than calculates the labeling index as the percentage of brown stained nuclear area over the total nuclear area. The system also produces a pseudo-colored result image, illustrating the area segmentation. Every generated image was checked for consistency by two pathologists (BR and SS). Only the correct segmented images were accepted for further analysis.

### Statistical methods

The follow-up endpoint for each patient represents a binary evaluation of the treatment response at 6 months after the end of the treatment. All existing factors were compared when examining the two groups of patients (CR and NCR).

Categorical factors were analyzed using a chi-squared test, and when reduced numbers of observations were present, we applied Yates’ correction [10]. A comparison of medians was performed using the median test and two-tailed unpaired t test was used to evaluate for differences in gene expression between groups of interes (NCR vs CR). The strengths of the association between genes of interest as well as between PCR and microarray results were tested with a Pearson parametric test. The receiver operating characteristic (ROC) curve was used to evaluate the predictive accuracy of genes of interest in the differentiation between samples with or without complete remission. [11]. The calculation of the area under curve (AUC) and test equality with a value of 0.5 was performed according to Bamber and Hanley [12,13]. The point of optimal classification was considered the point nearest to (0.1) of the absolute classification. Unpaired t-test on arcsine-transformed data was used to determine whether the proportion of stained nuclear protein was different between non-responders and responders samples, in both genomic and IHC validation groups.

All differences with p < 0.05 were considered statistically significant. The confidence intervals were evaluated with the level of significance equal to 0.05.

## Results

### Patient and tumor characteristics

FIGO staging evaluation of the patients included in this study revealed that approximately 48% of the patients were in stage II, while the rest of 52% were in stage III. Among these, 2 patients tested negative for HPV, whereas HPV-16 subtype has been detected in the majority of the cases. Based on 6 months treatment outcome evaluation twelve patients presented complete remission and were assigned to the CR group, while the rest of 9 patients that partially responded or had stable disease were assigned to the NCR group. We observed higher median age value in the responders group (p < 0.01), however prognostic factors such as tumor size, hemoglobin and FIGO stage were balanced between the NCR and CR groups (Table 2). Since almost all the patients presented HPV 16-positive tumors, the association between HPV subtype and treatment outcome could not be assessed.

**Table 2:** Association between clinical and histopathological data and treatment response

**Characteristics**	**No. of patients**	**CR group**	**NCR group**	**p**
	**n (%)**	**n**	**n**	
**Study population**	21 (100%)	12	9	
**Age (years)**				
Median	21	56.5	42	< 0.01
Range		28-73	27-55	
**Tumor size (cm)**				
Median	21	4.75	5	0.054
Range		2.0-6.5	2.5-8.0	
**Hemoglobin (g/dl)**				
Median	21	12.75	12.2	0.8
Range		7.9-14.2	10.1-14.4	
**FIGO stage**				
IIB	10 (47.62%)	4	6	0.62
IIIA	5 (23.81%)	4	1	
IIIB	6 (28.57%)	4	2	
**HPV subtype**				
16	16 (76.19%)	10	6	-
Other high-risk	3 (14.29%)	1	2	
Negative	2 (9.52%)	1	1	

### Gene expression profiling of cervical cancer samples

Gene expression profiles for NCR and CR samples were generated using one-color hybridization to whole human genome arrays carrying 43,376 biological sequences. We assessed the quality of the array before and after normalization and we did not detect batch effects or outlier arrays. We observed a weak correlation between background and foreground intensities across each array (r range, 0.06 to 0.2), therefore we did not perform background correction. To improve data quality, a filtering step was applied. A total number of 40,998 sequences passed the filtering criteria and were used for further analysis. In class comparison analysis we identified a signature of 2859 genes whose differential expression in non-responder compared to responder samples exceeded 1.5-fold at an adjusted p-value < 0.05. Of these, 1501 genes were up-regulated and 1358 genes were down-regulated in NCR compared with CR.

**Figure 1:**
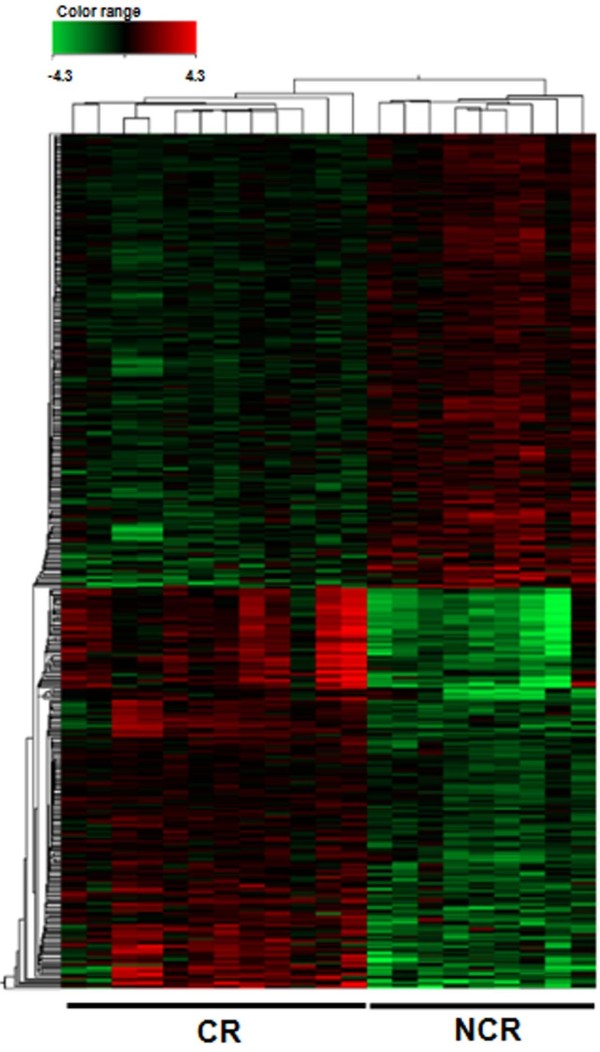
**Heatmap of differentially expressed genes between CR (n = 9) and NCR (n = 12) samples obtained from supervised hierarchical clustering using Euclidean distances and Ward method.** The color indicates the level of mRNA expression: red - higher level of expression, green - lower level of expression, black – no expression changes (each row represents a gene and each column represents a sample). The CR samples were clustered together and clearly separated from NCR samples.

To highlight the differences in gene expression a supervised hierarchical clustering was performed on the set of differentially expressed genes. Based on expression profiles, non-responder and responder samples were grouped in two distinct main clusters (Figure 1).

### Functional profile assessment

To obtain a global view of the altered biological functions and canonical pathways that could be responsible for intrinsic resistance in cervical cancer, we performed functional analysis in IPA. We chose to evaluate the biological functions and canonical pathways because it provides more robust results than studying individual genes. Sixty-five significant molecular functions have been predicted in IPA (p < 0.05) to be mediated by differentially expressed genes identified in NCR vs. CR samples. ‘Cellular Movement’ (p = 5.30E-08-1.22E-02) was the top biological function mediated by these genes followed by ‘Cell Cycle’ (p = 7.12E-07-1.22E-02) and ‘DNA Replication, Recombination and Repair’ (p = 7.12E-07-1.18E-02). The dataset of differentially expressed genes were also integrated in 34 canonical pathways. The ‘Role of BRCA1 in DNA Damage Response’ was predicted to be the most significantly activated canonical pathway (p = 1.86E-04), which suggests a baseline intrinsic resistance of non-responding cervical cancer tumors. The top five molecular and cellular functions and the canonical pathways with associated p-values are presented in Table 3.

**Table 3:** The top significant molecular and cellular functions identified by IPA

**Molecular and cellular functions**	**p value**	**No. genes**
Cellular movement	5.30E-08-1.22E-02	344
Cell cycle	7.12E-07-1.22E-02	233
DNA replication, recombination and Rrepair	7.12E-07-1.18E-02	124
Cellular development	1.16E-06-1.22E-02	433
Cellular assembly and organization	4.97E-06-1.22E-02	322
**Canonical pathways**	**p value**	**Ratio**
Role of BRCA1 in DNA damage response	1.86E-04	17/65 (0.262)
Primary immunodeficiency signaling	3.97E-03	11/62 (0.177)
G protein signaling mediated by Tubby	5.05E-03	9/42 (0.214)
Aryl hydrocarbon Rreceptor signaling	5.39E-03	25/161 (0.155)
Regulation of actin-based motility by Rho	7.5E-03	17/89 (0.191)

**Figure 2:**
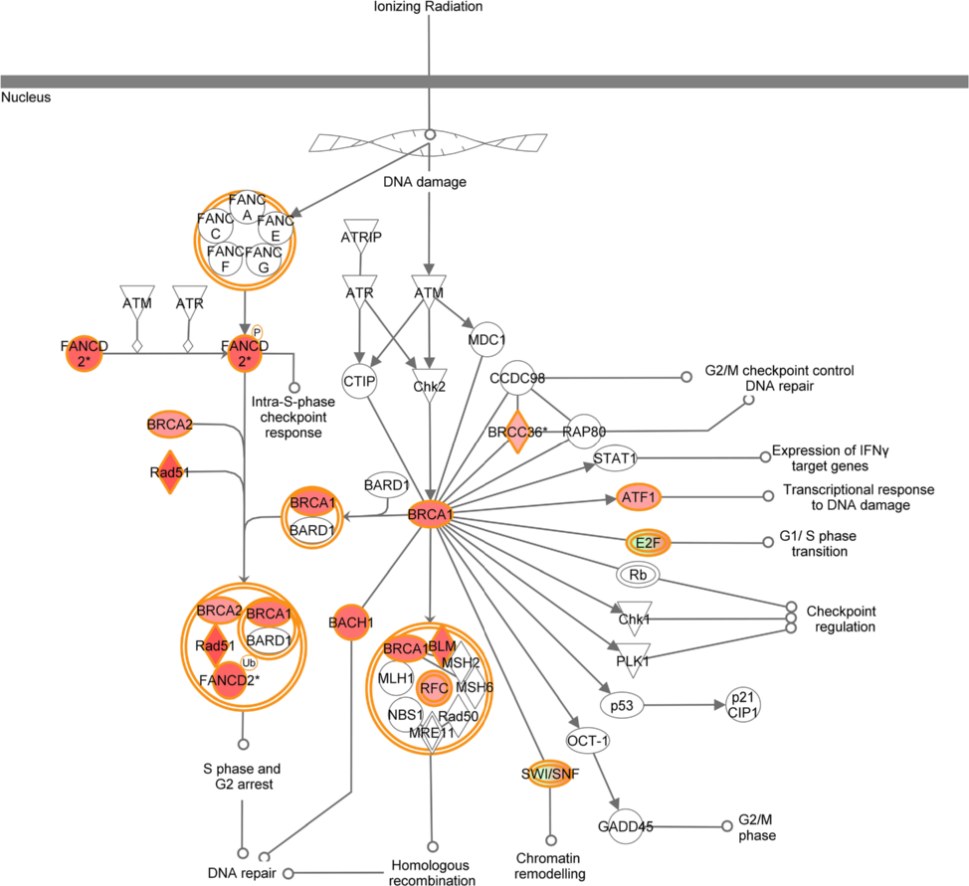
**Activation of the "Role of BRCA1 in DNA Damage Response’ pathways in NCR versus CR samples.** Genes highlighted in red were significantly overexpressed in non-responsive compared with responsive cervical cancers.

It is known that cancer becomes resistant to therapy by restoring the DNA repair machinery; therefore, we focused our attention on the genes involved in ‘DNA Replication, Recombination and Repair’ molecular mechanisms. In total, 124 genes from our dataset were listed in these mechanisms (Additional file 1). The vast majority of genes (n = 92) were overexpressed with fold change between 1.503 and 2.867 while 32 genes were down-regulated (fold change: -10.471 to -1.509) in NCR vs. CR cervical samples. Among these genes, seventeen (RAD51, BRIP1, BLM, BRCA1, BRCA2, BRCC3, HLTF, FANCD2, FANCI, FANCM, FANCL, ATF1, E2F4, E2F2, SMARCA2, SMARCA4 and RFC1) were significantly associated in IPA with the ‘Role of BRCA1 in DNA Damage Response’ pathway (p = 1.86E-04, ratio = 0.262) (Table 4). The overexpression of BRCA1, BRCA2, RAD51, BRIP1 (BACH1), FANCD2, BLM and RFC in non-responding versus responding cervical cancer samples suggests that DNA repair mechanism activation occurs through cell cycle arrest and homologous recombination (Figure 2).

**Table 4:** Genes involved in the "Role of BRCA1 in DNA Damage Response" pathway with associated p-values obtained from microarray experiment

**Ref seq**	**Gene symbol**	**Fold regulation (NCR vs CR)**	**Adjusted p-value**	**Description**
NM_005171	ATF1	1,747	0,013	activating transcription factor 1
NM_000057	BLM	2,430	0,030	Bloom syndrome, RecQ helicase-like
NM_007300	BRCA1	2,225	0,008	breast cancer 1, early onset
NM_000059	BRCA2	1,842	0,011	breast cancer 2, early onset
NM_001018055	BRCC3	1,621	0,016	BRCA1/BRCA2-containing complex, subunit 3
NM_032043	BRIP1	2,353	0,018	BRCA1 interacting protein C-terminal helicase 1
NM_004091	E2F2	2,137	0,048	E2F transcription factor 2
NM_001950	E2F4	-1,791	0,013	E2F transcription factor 4, p107/p130-binding
NM_001018113	FANCB	2,216	0,010	Fanconi anemia, complementation group B
NM_033084	FANCD2	1,613	0,012	Fanconi anemia, complementation group D2
NM_018062	FANCL	1,701	0,031	Fanconi anemia, complementation group L
NM_020937	FANCM	1,923	0,005	Fanconi anemia, complementation group M
NM_139048	HLTF	2,245	0,016	helicase-like transcription factor
NM_002875	RAD51	2,767	0,010	RAD51 homolog (S. cerevisiae)
NM_002913	RFC1	1,723	0,004	replication factor C (activator 1) 1, 145 kDa
NM_139045	SMARCA2	1,659	0,041	SWI/SNF related, matrix associated, actin dependent regulator of chromatin, subfamily a, member 2
NM_003072	SMARCA4	-1,519	0,026	SWI/SNF related, matrix associated, actin dependent regulator of chromatin, subfamily a, member 4

### qRT-PCR validation of the microarray results

**Figure 3:**
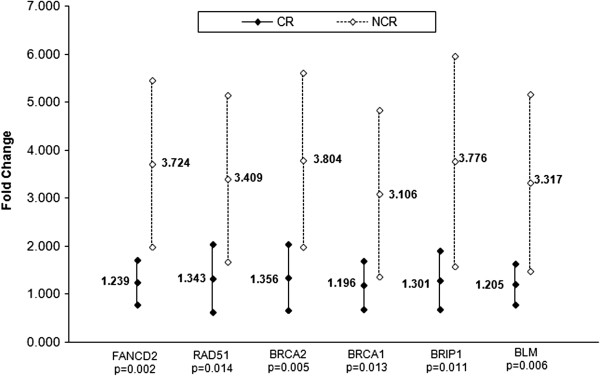
**qRT-PCR validation data for six genes (FANCD2, RAD51, BRCA2, BRCA1, BRIP1/BCH1 and BML) involved in the ‘Role of BRCA1 in DNA Damage Response’ pathway.** Fold change was calculated using the ΔΔCt method relative to the CR group.

In order to assess the accuracy of microarray results, six genes including RAD51, BRIP1 (BACH1), BRCA1, BRCA2, BLM and FANCD2 involved in the ‘Role of BRCA1 in DNA Damage Response’ pathway were selected for validation by qRT-PCR. The fold changes calculated between NCR vs. CR samples revealed at least 3-fold up-regulation for all genes of interest (Figure 3). We assessed the correlation between the qRT-PCR and microarray results by computing Pearson’s correlation coefficients for each gene. A strong correlation between the two methods was observed (r = 0.705 - 0.835) (Table 5).

**Table 5:** **Pearson’s correlation coefficients of log**_**2 **_**fold change values obtained from microarray and PCR experiments**

**Gene**	**Pearson coefficient**	**p**
BLM	0.835	< 0.0001
BRIP1	0.811	< 0.0001
BRCA1	0.765	< 0.0001
BRCA2	0.721	0.0002
RAD51	0.705	0.0005
FANCD2	0.759	< 0.0001

### Assessment of the prognostic significance of genes involved in ‘Role of BRCA1 in DNA Damage Response’ pathway

We estimated the prognostic significance of the six selected genes by the ROC analysis. We analyzed the ROC curves for all previously known potential factors, including age, tumor size, hemoglobin, along with our potential markers: BRCA1, BRCA2, RAD51, FANCD2, BLM and BRIP1. If the p-value was not significant (p > 0.05), then the AUC, sensitivity, specificity and optimal classification point were omitted. The investigated genes discriminated between the patients in the NCR and CR groups (p < 0.01) suggesting a superior predictive value compared to classical factors such as tumor size and hemoglobin. The summary of the ROC curves (AUC, specificity and sensitivity) for all six genes is presented in Table 6.

**Table 6:** ROC analysis for prognostic factors

**Nr.crt.**	**Variable**	**AUC**	**Classification point**	**Sensitivity**	**Specificity**	**p**
1.	BRCA1	0.81	<0.858565	0.92	0.78	<0.01
2.	BRCA2	0.86	<0.602903	0.75	0.89	<0.01
3.	RAD51	0.81	<0.895025	0.91	0.78	<0.01
4.	FANCD2	0.84	<0.673616	0.83	0.78	<0.01
5.	BLM	0.81	>0.871154	0.63	0.99	<0.01
6.	BRIP1	0.81	>0.606256	0.78	0.75	<0.01
7.	Age (years)	0.86	>46	0.75	0.89	<0.01
8.	Tumor size (cm)	0.65	-	-	-	0.11 (NS)
9.	Hb (g/dl)	0.64	-	-	-	0.39 (NS)

**Figure 4:**
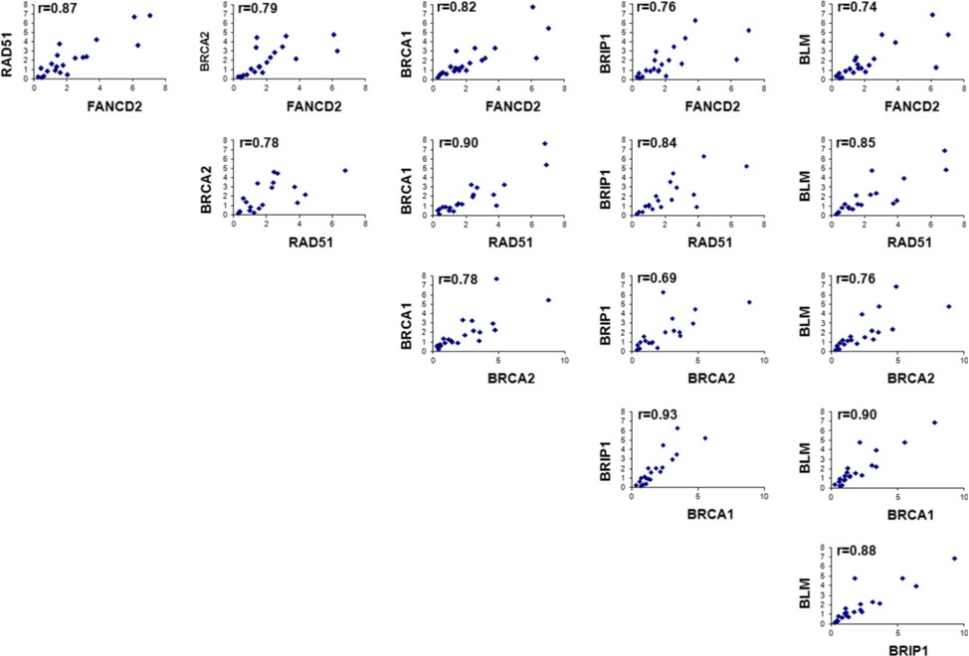
Pearson correlations between fold change values of the target genes.

The correlations between the target genes BRCA1, BRCA2, RAD51, FANCD2, BLM and BRIP1 indicated that all genes were highly correlated with each other. The correlation coefficients were between 0.69 (BRCA2 vs. BRIP1) and 0.93 (BRCA1 vs. BRIP1) (Figure 4).

### IHC validation of the microarray results

**Figure 5:**
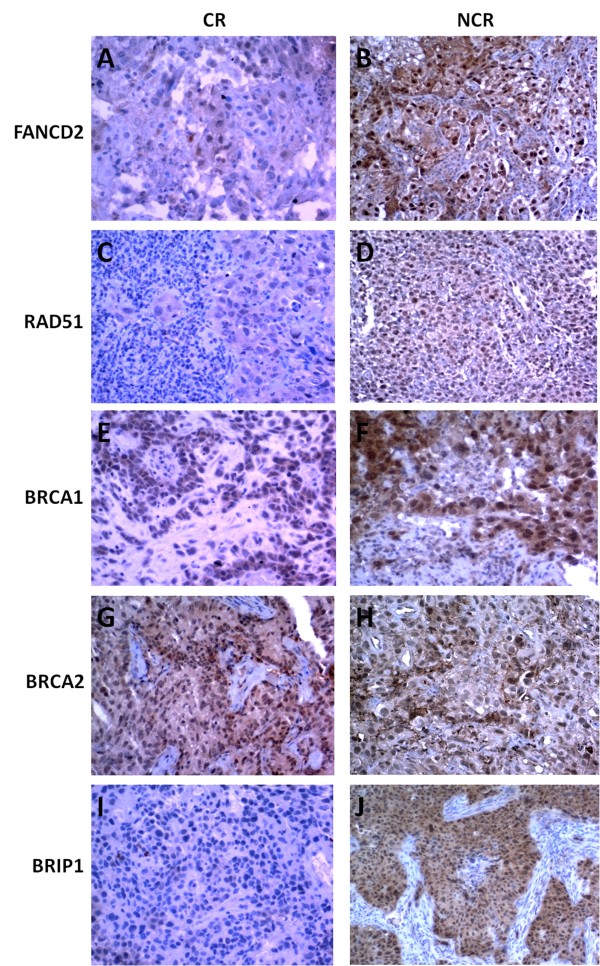
**Validation of FANCD2 (A-B), RAD51 (C-D), BRCA1 (E-F), BRCA2 (G-H) and BRIP1 (I-J) protein expression in advanced squamous cervical tumor cells.** Staining for FANCD2, RAD51, BRCA1 and BRIP1 for the NCR cervical samples indicated strongly positive protein expression compared with the CR cervical samples. The BRCA2 protein expression was comparable between the NCR and CR cervical samples; (x200 magnification).

Immunohistochemical staining was performed to obtain further validation of microarray findings. We assessed the protein expression of RAD51, BRIP1 (BACH1), BRCA1, BRCA2, BLM and FANCD2 in all 21 samples used in the genomic study (Figure 5). For BLM gene we did not identified a specific monoclonal antibody (MoAb), therefore this gene could not be taken into account for protein validation. An average percentage of nuclear staining on 3 different representative images of every sample was calculated for every protein of interest. We observed a significantly increased protein levels of FANCD2, BRCA1, RAD51 and BRIP1 in the nuclei of the NCR compared to the CR cervical tumors. No difference was observed for nuclear protein expression of BRCA2 in NCR compared to CR tissues. A ratio between nuclear protein expressions in NCR and CR groups was calculated (Table 7).

**Table 7:** Assessment of the nuclear proteins including FANCD2, RAD51, BRCA2, BRCA1, BRIP1(BACH1) in NCR and CR cervical tumors evaluated both for genomics study set (n = 21) and IHC validation set (n = 24)

**Proteins**	**Cellular localization**	**NCR %**	**CR %**	**NCR/CR ratio**	**p-value**
**Genomics study set (n = 21)**					
FANCD2	nucleus	32.53	15.6	2.09	**0.032**
RAD51	nucleus	14.88	7.11	2.09	**0.016**
BRCA2	nucleus	21.11	20.81	1.01	0.868
BRCA1	nucleus	27.78	10.69	2.60	**0.032**
BRIP1 (BACH1)	nucleus	40.44	10.05	4.02	**0.001**
**IHC validation set (n = 24)**					
FANCD2	nucleus	25.34	16.22	1.56	**0.011**
RAD51	nucleus	16.87	4.86	3.47	**0.000**
BRCA1	nucleus	27.59	12.97	2.13	**0.000**
BRIP1 (BACH1)	nucleus	30.73	14.55	2.11	**0.011**

The p-values in the bold format are statistically significant.

An additional set of 24 FFPE squamous cervical samples (15 CR and 9 NCR) was used as an independent validation of the protein data. Increased protein levels of FANCD2, RAD51, BRCA1, and BRIP 1 (BACH1) in NCR compared to CR cervical tumors groups were confirmed on the validation set (Table 7).

## Discussion

Cervical cancer continues to represent a major health problem for women from developing countries. Cervical cancer lethality occurs because most patients are first diagnosed in advanced stages. Even if early stages are successfully treated, advanced cervical cancer represents a major problem due to increased rates of recurrence and distant metastasis. Although knowledge about tumor biology and various mechanisms of resistance has increased in recent years, different schedules of treatment, including new anticancer drugs, have not efficiently reduced the occurrence of drug resistance. Intrinsic resistance is often decisive for treatment failure; almost half of patients present with baseline resistance, rendering classical therapies ineffective.

In an effort to elucidate the patterns of genes involved in baseline resistance, we performed a genome-wide microarray assay on primary biopsies from patients with advanced cervical cancers with known clinical and histological responses. All of the patients included in the study received radiotherapy as the main therapy and cisplatin as a radiosensitizer Based on the microarray analysis, we identified a supervised gene expression profile that differed dramatically between the non-responding and responding cervical tumors. ‘DNA Replication, Recombination and Repair’ represents one of the most important molecular patterns identified as important for intrinsic resistance in cervical cancer. In our study, the non-responding cervical tumor cells had more active DNA damage repair machinery than responding cervical tumor cells, even before starting the therapy. In total, 92 out of the 124 identified genes involved in ‘DNA replication, recombination and repair’ were overexpressed in the non-responding tumors compared with the responding tumors (Additional file 1).

Cancer cells become resistant to therapy by restoring DNA repair genes; therefore, we looked for pathways involved in the maintenance of DNA stability. By classifying the genes according to functional pathways, we identified the ‘Role of BRCA1 in DNA Damage Response’ as the most important canonical pathway involved in DNA repair (Table 3). To our knowledge, there are no studies that describe ‘Role of BRCA1 in DNA Damage Response’ pathway as predictive for treatment outcome in cervical cancer, even though a conserved pathway for increased DNA repair mediated by BRCA1 was described for other pathologies [14,15]. Among the genes significantly up-regulated in the BRCA1 canonical pathway, we focused our attention on a set of six genes that were considered of particular interest: BRCA1, BRCA2, RAD51, FANCD2, BACH1/BRIP1/FANCJ and BLM. The expression of these genes detected by microarray was confirmed by qRT-PCR with good correlation (Table 5).

Early studies on BRCA1 and BRCA2 have reveled that both proteins are involved in DSB repair. In this study, we showed that BRCA1 and BRCA2 overexpression in patients with advanced cervical cancer is associated with treatment failure. Several studies have pointed out that BRCA-deficient cells are inefficient at repairing DNA damage by homologous recombination (HR) [16,17] and are thus more sensitive to chemotherapeutic drugs. Zhang et al. [18] reported that the E6 and E7 HPV oncoproteins interact with BRCA1 and alter its activity in cervical cancer cells. However, the association between high-risk HPV genotypes and treatment failure could not be evaluated in our study as our sample set did not comprise a sufficient number of other high-risk types. Recently, a so-called BRCAness gene expression profile has also been correlated with response to chemotherapy and outcome in patients with epithelial ovarian cancer [19]. BRCA1 is a component of the BASC complex that is important for efficient DNA repair. MSH2/MSH6, PMS2/MLH1, BLM helicase and the replication factor C (RFC) represent other important members of the BASC complex [20].

Our microarray data pointed out an increased level of BLM and RFC1 in the non-responding cervical cancers compared with the responding cancers. Additionally, BRCA1 associates with the SWI/SNF chromatin-remodeling complex and FANCD2 [21] and plays a role in regulating the cellular localization of BACH1/BRIP1 (BRCA1-associated carboxyl-terminal helicase). BRCA2 is also involved in DNA repair; the protein interacts specifically with RAD51, an essential protein involved in HR [22]. In our efforts to understand the molecular basis of treatment response in advanced cervical cancer, besides the BRCA pathway, we also found the fanconi anemia (FA) complementation group, FANCD2, FANCL, FANCM, FANCJ/BRIP1/BACH and FANCI, to be involved in intrinsic resistance to chemo-radiotherapy. These FA proteins are closely related to the BRCA1 and BRCA2 gene products and their partner proteins and are required for cellular resistance to agents that cause DNA interstrand cross-links (ICLs) [23]. The FANCD2 protein colocalizes to nuclear foci together with BRCA1, BRCA2 and RAD51 and initiates homology-directed DNA repair in a "FA/BRCA pathway,’ both in response to DNA-damaging agents (cisplatin, ionizing radiation, hydroxyurea, etc.) and in the absence of exogenous DNA damage during the S phase of the cell cycle [24].

Our results revealed an increased protein level of FANCD2, RAD51, BRCA1 and BRIP1 in the NCR compared to CR cervical tumor nuclei. These observations were also confirmed on an independent validation set, emphasizing the role of these four proteins in CRT resistance (Table 7). Although we observed a 3.8-fold increase in BRCA2 mRNA in NCR vs. CR cervical samples (qRT-PCR data), there was no significant difference for BRCA2 protein between NCR and CR groups, which could be due to either using an inadequate monoclonal antibody clone or posttranscriptional modifications of the BRCA2 transcript. A central step in the FA/BRCA pathway is the monoubiquitylation of FANCD2 and its translocation to chromatin at the site of DNA damage [25]. The ubiquitylation of FANCD2 is initiated by FANCM and is mediated by the UBE2T (E2) enzyme and a multisubunit ubiquitin E3 ligase that consists of eight FA proteins (FANCA/B/C/E/F/G/L/M) [26]. FANCD2 can also be monoubiquitylated and chromatin-loaded by the E3 ubiquitin ligase activity of RAD18 in a FA-independent manner [27].

Increased expression of FANCM, FANCL, UBE2T and RAD18 mRNA was observed in NCR compared to CR cervical tumor samples (Additional file 1). We also observed a slight increase in SLX4/FANCP/BTBD12 mRNA (fold change = 1.3, p = 0.035) for the NCR vs. CR cervical samples. SLX4 is a novel member of the FA genes that coordinates multiple DNA repair pathways by acting as a scaffold for multiple nucleases involved in ICL repair and the mechanisms involved in HR [28]. Depletion of SLX4 leads to hypersensitivity to cisplatin and reduced efficiency of HR repair [29]. Narayan et al. [30] showed that advanced cervical cancer is associated with alterations in the FA/BRCA pathway by either promoter hypermethylation and/or deregulated gene expression compared with the normal cervix. FA inhibitors were recently proposed as important tools for overcoming cisplatin resistance in tumors [31].

RAD51, one of the key molecules of DNA repair, is another gene we found to be significantly overexpressed in the NCR cervical cancers. We observed a 3.4-fold increase in RAD51 mRNA (qRT-PCR data) and also an increased protein expression of RAD51 in the nuclei of the NCR vs. CR cervical cancers. RAD51 has anti-apoptotic activity in tumor cells [32], and high expression of this protein is correlated with poor prognosis, resistance to ionizing radiation and drug resistance [33]. RAD51 is essential in the HR process of DNA repair, its expression being tightly controlled in normal healthy cells to maintain genomic stability [34]. RAD51 co-localizes with BRCA1 and FANCD2 in S-phase specific nuclear foci and initiates homology-driven repair activity. Several studies have shown that in cancer cells, this molecule is overexpressed, leading to radio- and chemoresistance [35,36]. Elevated levels of RAD51 have been associated with increased invasiveness in breast cancer patients [37] and have been demonstrated to be an independent prognostic marker of survival in non-small cell lung cancer patients [38]. A previous microarray study of paired cervical tumor samples (pre- and post-chemoradiotherapy) reported down-regulation of RAD51 after treatment [5], supporting the hypothesis that radiation sensitivity is facilitated by a diminished DNA repair response. Targeting strategies against this gene have been developed as possible anticancer treatments; attempts to inhibit RAD51 have proven to be successful in reducing treatment resistance in tumor cells [39,40].

In a previous study, it was shown that poly(ADP-ribose) polymerase (PARP) inhibitors could suppress the expression of BRCA1 and RAD51 [41]. The PARP family, especially PARP1 and PARP2, functions as DNA damage sensors and recruits a variety of DNA repair proteins to the site of damaged DNA [42]. In BRCA-positive breast cancer, PARP inhibitors were found to increase the cytotoxic effects of radiation and chemotherapy based on the principle of synthetic lethality [43]. In our microarray study, we noted a higher level of PARP2 mRNA in the NCR vs. CR cervical cancer samples (Additional file 1).

Previous studies have shown that prognostic factors including younger age [44], tumor size [45], anemia (hemoglobin) and FIGO stage [46], are used to estimate overall survival, disease-free survival and local control in cervical cancer. Nevertheless, they not provide information about the baseline resistance and the tumor heterogeneity. Our data indicates age as significant factor for treatment response, however the sample size is limited and no final conclusions can be drawn. Taking into account our findings and the need to identify new valuable prognostic factors for baseline resistance, we suggest that if our results are confirmed on a larger study, the assessment of nuclear expression of FANCD2, RAD51, BRCA1 and BRIP1 proteins could represent a supplementary prognostic factor that would better tailor the treatment for patients with advanced cervical cancer. These results could be the foundation for the development of new targeting strategies to improve cervical cancer outcome.

## Conclusions

Our data revealed high DNA repair machinery activity even before starting radio-chemotherapy in NCR patients compared with CR patients. Therefore, our findings demonstrate that baseline FANCD2, RAD51, BRCA1 and BRIP1 nuclear protein expression could have an important role in treatment failure in advanced squamous cervical cancer. To our knowledge, this is the first study to demonstrate the role of the FA/BRCA pathway in baseline resistance and therapy failure in locally advanced cervical cancer.

### Limitations

The limitation of this study is related to the small number of samples even if we used an independent validation set for protein data to increased confidence of these findings. Larger studies have to confirm that the assessment of these proteins could represent an important prognostic factor that determines poor response to radiation and chemotherapy for locally-advanced cervical cancers.

## Abbreviations

AUC: Area under curve; BT: Brachytherapy; CR: Complete response; CRT: Concomitant chemoradiotherapy; Cy3: Cyanine 3; DAB: Diamino-benzidine tetrachloride; DSBs: DNA double-strand breaks; EBRT: External beam radiotherapy; FDR: False discovery rate; FIGO: International Federation of Gynecology and Obstetrics; HDR: High-dose-rate; HPV: Human papillomavirus; IHC: Immunohistochemistry; IPA: Ingenuity pathway analysis; NCR: Non-complete response; RIN: RNA integrity number; ROC: Receiver operating characteristic.

## Competing interests

The authors declare that there are no competing interests.

## Authors’ contributions

OB designed and coordinated the research study, performed the microarray experiment, interpreted the results and drafted the manuscript; LB performed bioinformatic analysis of microarray data and drafted the manuscript; OT performed qRT-PCR analysis and revised the manuscript; NT performed statistical analysis; MR and SV processed the samples for microarray analysis; RB and SS assessed the IHC staining; BF performed histopathological evaluation of the samples; LP and LM processed the tissues for IHC staining; CO enrolled and followed up patients, IBN interpreted the results and revised the manuscript, VN enrolled and followed up patients and revised the manuscript. All authors read and approved the final manuscript.

## Pre-publication history

The pre-publication history for this paper can be accessed here:

http://www.biomedcentral.com/1471-2407/14/246/prepub

## Supplementary Material

Additional file 1 Genes involved in DNA replication, recombination, and repair mechanisms. Click here for file
